# Predictive ability of pharyngeal inlet angle for the occurrence of postoperative dysphagia after occipitocervical fusion

**DOI:** 10.1186/s12891-020-03921-y

**Published:** 2021-01-09

**Authors:** Lin-nan Wang, Bo-wen Hu, Yue-ming Song, Li-min Liu, Chun-guang Zhou, Lei Wang, Xi Yang

**Affiliations:** Department of Orthopedics Surgery and Orthopaedics Research Institute, West China Hospital, Sichuan University, No. 37 GuoXue Road, 610041 Chengdu, Sichuan China

**Keywords:** Occipitocervical fusion, Postoperative dysphagia, O-EAa, PIA, Prediction

## Abstract

**Background:**

PIA has been proven to be a predictor for postoperative dysphagia in patients who undergo occipitospinal fusion. However, its predictive effect for postoperative dysphagia in patients who undergo OCF is unknown. The aim of this study was to evaluate the predictive ability of the pharyngeal inlet angle (PIA) for the occurrence of postoperative dysphagia in patients who undergo occipitocervical fusion (OCF).

**Methods:**

Between 2010 and 2018, 98 patients who had undergone OCF were enrolled and reviewed. Patients were divided into two groups according to the presence of postoperative dysphagia. Radiographic parameters, including the atlas-dens interval (ADI), O-C2 angle (O-C2a), occipital and external acoustic meatus to axis angle (O-EAa), C2 tilting angle (C2Ta), C2-7 angle (C2-7a), PIA and narrowest oropharyngeal airway space (nPAS), were measured and compared. Simple linear regression and multiple regression analysis were used to evaluate the radiographic predictors for dysphagia. In addition, we used PIA = 90° as a threshold to analyze its effect on predicting dysphagia.

**Results:**

Of the 98 patients, 26 exhibited postoperative dysphagia. Preoperatively, PIA in the dysphagia group was significantly higher than that in the nondysphagia group. We detected that O-C2a, O-EAa, PIA and nPAS all decreased sharply in the dysphagia group but increased slightly in the nondysphagia group. The changes were all significant. Through regression analyses, we found that PIA had a similar predictive effect as O-EAa for postoperative dysphagia and changes in nPAS. Additionally, patients with an increasing PIA exhibited no dysphagia, and the sensitivity of PIA <90° in predicting dysphagia reached 88.5%.

**Conclusions:**

PIA could be used as a predictor for postoperative dysphagia in patients undergoing OCF. Adjusting a PIA level higher than the preoperative PIA level could avoid dysphagia. For those who inevitably had decreasing PIA, preserving intraoperative PIA over 90° would help avert postoperative dysphagia.

**Trial registration:**

This trial has been registered in the Medical Ethics Committee of West China Hospital, Sichuan University. The registration number is 762 and the date of registration is Sep. 9 th, 2019.

## Background

Dysphagia is one of the most commonly seen complications after occipitocervical fusion (OCF), with an incidence ranging from 15.8–26.6% [1–4]. Most of these patients have a protracted course, and dysphagia interferes with the quality of daily living [3–6]. For this reason, the relationship between postoperative dysphagia and changes in occipitocervical alignment has been studied by many researchers, and several predictors have been promoted [1, 3–5, 7, 8].

The O-C2 angle (O-C2a) was the most commonly used predictor in previous studies since it was first described in 2007 [9]. A significant reduction in O-C2a was correlated with a decrease in oropharyngeal space and postoperative dysphagia [1, 10, 11]. The newly promoted occipital and external acoustic meatus-to-axis angle (O-EAa) is the sum of the O-C2a and C2 tilting angles (C2Ta) and can reflect not only craniocervical junction alignment but also cranial transverse motion, thus compensating for the shortcomings of O-C2a [7]. In our previous study, we reviewed 109 OCF patients and found that O-EAa was superior to O-C2a in predicting decreases in oropharyngeal space and postoperative dysphagia [4]. However, during the measurement of O-EAa, we found it difficult to identify the external acoustic meatus in some patients.

The pharyngeal inlet angle (PIA) was promoted by Kaneyama in 2017 and was defined as the angle between McGregor’s line and the line that links the center of the C1 anterior arch and the apex of cervical sagittal curvature [3]. These anatomical structures could be easily identified on intraoperative radiographic images. Previously, this angle has been proven to be a predictor for postoperative dysphagia in patients who undergo occipitospinal fusion [3]. Therefore, we conducted this study to evaluate the predictive effect of PIA for the development of postoperative dysphagia in patients who undergo OCF and to compare the predictive abilities of PIA and several other radiographic parameters.

## Methods

In the present study, we reviewed the same batch of patients from April 2010 to June 2018 as in our previous study [4]. The inclusion criteria were as follows: (1) post-OCF patients; (2) a minimum follow-up of 12 months; and (3) complete preoperative and postoperative radiographic records. The exclusion criteria were as follows: (1) presence of preoperative dysphagia; (2) underwent transoral surgery or esophageal disease; (3) unable to evaluate postoperative swallowing conditions; and (4) unidentifiable anterior arch of C1 on lateral radiographic images.

### Radiographic measurements

**Fig. 1:**
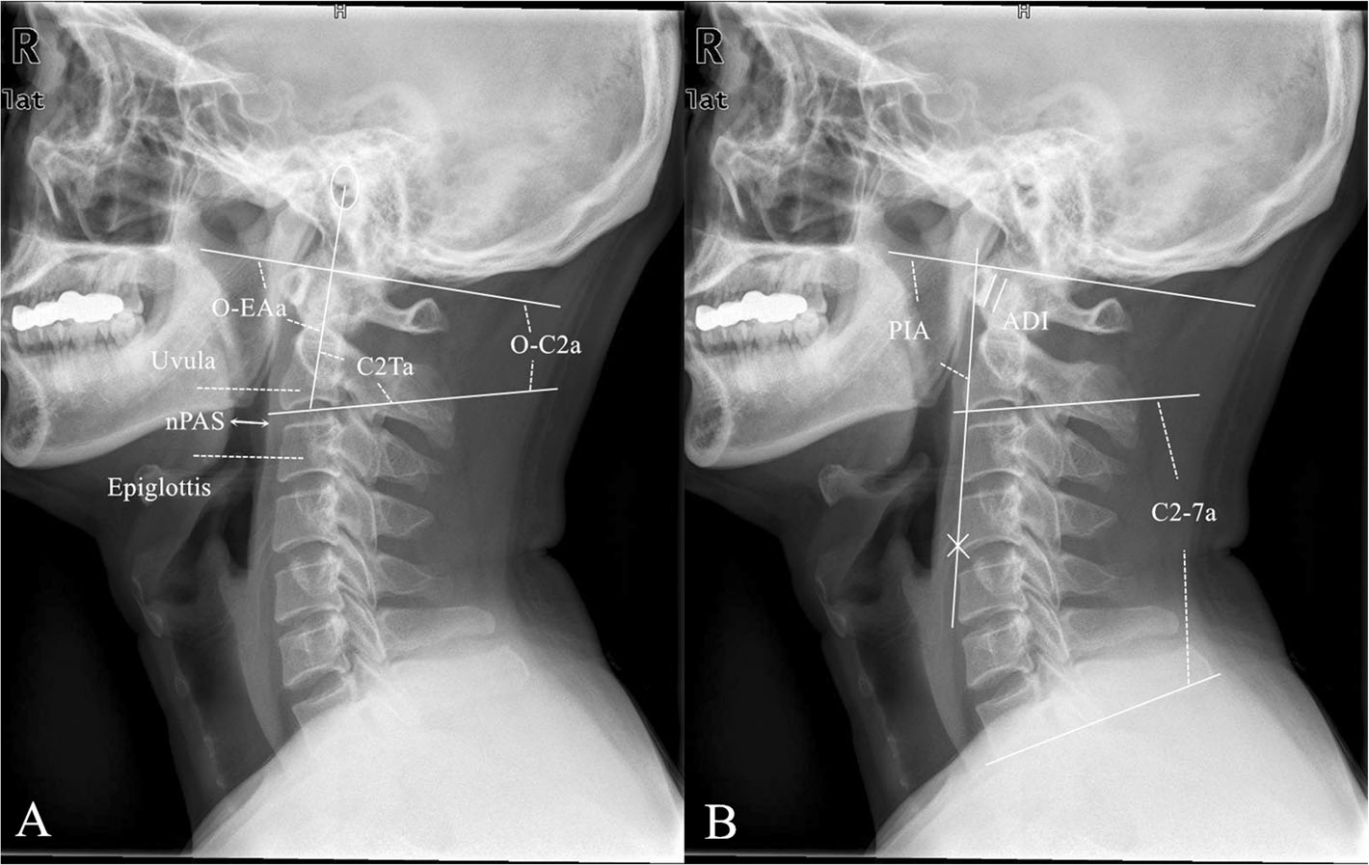
Representation of radiographic measurements. **a** O-C2a is the angle between McGregor’s line and the inferior endplate of the axis. O-EAa is the angle formed by McGregor’s line and the line connecting the midpoint of the bilateral external acoustic meatus and the midpoint of the inferior end plate of C2 (EA line). C2Ta is the angle formed by the inferior endplate of the C2 and EA lines. nPAS is the narrowest anterior-posterior diameter of the oropharynx between the tips of the uvula and epiglottis. **b** ADI is the distance between the back edge of the anterior arch of the atlas and the front edge of the odontoid process. C2-7a is the angle between the inferior endplate of C2 and C7. PIA is the angle between McGregor’s line and the line that links the center of the C1 anterior arch and the apex of cervical sagittal curvature

Preoperative and one month postoperative standing lateral cervical radiographic images in a neutral position with a horizontal gaze were used to measure the following radiographic parameters (Fig. 1):

Atlas-dens interval (ADI)- the distance between the back edge of the anterior arch of the atlas and the front edge of the odontoid process;

O-C2a- the angle between McGregor’s line and the inferior end plate of C2;

O-EAa- the angle formed by McGregor’s line and the line connecting the midpoint of the bilateral external acoustic meatus and the midpoint of the inferior end plate of C2 (EA line);

C2Ta- the angle formed by the inferior endplate of C2 and the EA line;

C2-7 angle (C2-7a)- the angle between the inferior endplate of C2 and C7;

PIA- the angle between McGregor’s line and the line that links the center of the C1 anterior arch and the apex of the cervical sagittal curvature;

Narrowest oropharyngeal airway space (nPAS)- the narrowest anterior-posterior diameter of the oropharynx between the tips of the uvula and epiglottis.

dADI, dO-C2a, dO-EAa, dC2Ta, dC2-7a, dPIA and dnPAS were calculated as the 1-month postoperative values minus the preoperative values. dnPAS% was calculated as dnPAS/preoperative nPAS.

### Dysphagia assessment

Inpatient medical records were reviewed to assess preoperative swallowing conditions. As in our previous study, the presence and duration of postoperative dysphagia were evaluated through inquiry by clinic or telephone interviews. The presence of postoperative dysphagia was defined as the occurrence of difficulty or taking extra effort in swallowing after surgery [12]. The grade of dysphagia was also evaluated for all patients during follow-up according to the dysphagia classification scale created by Bazaz et al. [13]. “Severe” dysphagia was defined as frequent difficulty swallowing with the majority of foods, “moderate” dysphagia was defined as occasional swallowing difficulty for specific foods, “mild” dysphagia was defined as rare episodes of dysphagia, and “none” was defined as no episodes of dysphagia. Patients with postoperative dysphagia less than one month duration was considered as side-effect caused by intubation and were considered as no occurrence of dysphagia.

### Statistical analysis

Measurements were independently performed twice in a week by two authors. Reliability was evaluated by calculating the intraclass correlation coefficient. Data were expressed as the mean ± standard deviation values. SPSS 23.0 (SPSS Inc, Chicago, Illinois) software was used to analyze the collected data. The paired Student’s *t* test and Mann-Whitney U test were used to compare continuous variables. A simple linear regression was used to investigate the association between collected radiographic parameters and the occurrence of postoperative dysphagia. Previously, we proved a positive correlation between nPAS% and dysphagia. Thus, we analyzed the association between nPAS% and other radiographic parameters. The correlation between dPIA and its potential influencing factors was also evaluated. A multiple regression analysis was used to examine the radiographic variables that affected dnPAS%. A p-value less than 0.05 indicated a significant difference. Power analysis (using 0.8 power and 0.05 significance) was used to calculate the required sample size.

## Results

Ninety-eight patients matched to the criteria were enrolled in this study, including 47 male patients and 51 female patients. The average age at the time of surgery was 50.7 years, and the mean follow-up period was 56.4 months (ranging from 12 to 110 months). Of all the patients, 26 experienced postoperative dysphagia, and the rate of occurrence was 26.5%. Among the 26 patients, only two experienced total relief of dysphagia during follow-up. However, dysphagia symptoms for the remaining 24 patients persisted until the final follow-up. Seven of these patients experienced partial relief. None of the 26 dysphagia patients underwent revision surgery. Dysphagia information of these patients at postoperative and final follow-up is shown in Table 1.

**Table 1:** Dysphagia information of patients in dysphagia group

No.	Grade of dysphagia (postoperative)	Duration (mo)	Grade of dysphagia (final follow-up)	No.	Grade of dysphagia (postoperative)	Duration (mo)	Grade of dysphagia (final follow-up)
1	Moderate	110	Mild	14	Moderate	43	Mild
2	Severe	108	Severe	15	Moderate	41	Moderate
3	Mild	98	Mild	16	Mild	41	Mild
4	Mild	8 (relief)	None	17	Mild	40	Mild
5	Moderate	74	Moderate	18	Moderate	24 (relief)	None
6	Mild	74	Mild	19	Mild	35	Mild
7	Severe	70	Severe	20	Severe	33	Moderate
8	Mild	68	Mild	21	Mild	20	Mild
9	Mild	66	Mild	22	Mild	18	Mild
10	Moderate	66	Mild	23	Moderate	16	Mild
11	Severe	55	Moderate	24	Moderate	13	Mild
12	Mild	48	Mild	25	Moderate	12	Moderate
13	Mild	46	Mild	26	Mild	12	Mild

### Radiographic results

The inter- and intraobserver intraclass correlation coefficient values for the radiographic parameters were all more than 0.9, demonstrating excellent repeatability.

We subdivided the patients into a dysphagia group and a nondysphagia group. Between the two groups, all parameters preoperatively were at a similar baseline except PIA. The preoperative PIA in the dysphagia group was significantly higher than that in the nondysphagia group. When comparing the postoperative values, we found that postoperative O-C2a, O-EAa, PIA and nPAS were significantly lower in the dysphagia group than in the nondysphagia group. However, C2-7a was significantly higher in the dysphagia group. In the dysphagia group, significant decreases were found in ADI, O-C2a, O-EAa, PIA and nPAS from preoperative to postoperative. In the nondysphagia group, we detected significant increases in O-C2a and decreases in ADI, C2Ta and C2-7a. The changes in O-C2a, O-EAa, C2-7a, PIA and nPAS% between the two groups all showed significant differences. Data are shown and compared in Table 2.

**Table 2:** Patients’ radiographic outcomes (mean ± SD)

	Dysphagia (26 patients)	Without Dysphagia (72 patients)	*P* value
**Pre ADI**	0.60 ± 0.26 cm	0.59 ± 0.35 cm	0.554
**Pre O-C2a**	9.58 ± 10.21	4.04 ± 16.01	0.191
**Pre O-EAa**	104.32 ± 7.44	101.33 ± 7.94	0.169
**Pre C2Ta**	94.73 ± 13.52	97.42 ± 17.72	0.504
**Pre C2-7a**	20.48 ± 14.06	25.67 ± 18.50	0.211
**Pre PIA**	96.80 ± 8.00	90.06 ± 11.42	0.005*
**Pre nPAS**	1.25 ±0.45cm	1.34±0.48cm	0.316
**Post ADI**	0.33±0.14cm ^#^	0.33±0.12cm^#^	0.971
**Post O-C2a**	0.07±9.12^#^	7.86±13.96^#^	0.005*
**Post O-EAa**	94.21±7.87^#^	101.42±6.44	0.000*
**Post C2Ta**	94.13±12.31	93.56±14.34^#^	0.863
**Post C2-7a**	24.60±13.27	18.00±15.58^#^	0.038*
**Post PIA**	81.85±5.51^#^	91.28±10.50	0.000*
**Post nPAS**	0.69±0.31cm^#^	1.43±0.50cm	0.000*
**dADI**	-0.27±0.22cm	-0.26±0.28cm	0.621
**dO-C2a**	-9.51±5.18	3.82±11.71	0.000*
**dO-EAa**	-10.11±5.12	0.10±7.15	0.000*
**dC2Ta**	-0.61±5.88	-3.86±11.37	0.159
**dC2-7a**	4.11±13.93	-7.66±13.57	0.001*
**dPIA**	-13.95±6.87	1.22±11.12	0.000*
**dnPAS%**	-43.84±16.33%	16.90±59.10%	0.000*

*SD* Standard deviation, *Pre* Preoperative, *ADI *Atlas-dens interval, *O-C2a *O-C2 angle, *O-EAa *Occipital and external acoustic meatus to axis angle, *C2Ta *C2 tilting angle, *PIA *Pharyngeal inlet angle, *nPAS *The narrowest oropharyngeal airway space, *Post* Postoperative, *d* The difference between pre- and postoperative values, *dnPAS%* (postoperative nPAS- preoperative nPAS)/preoperative nPAS*p<0.05 between groups; ^#^*p*<0.05 comparing with preoperative data

**Fig. 2:**
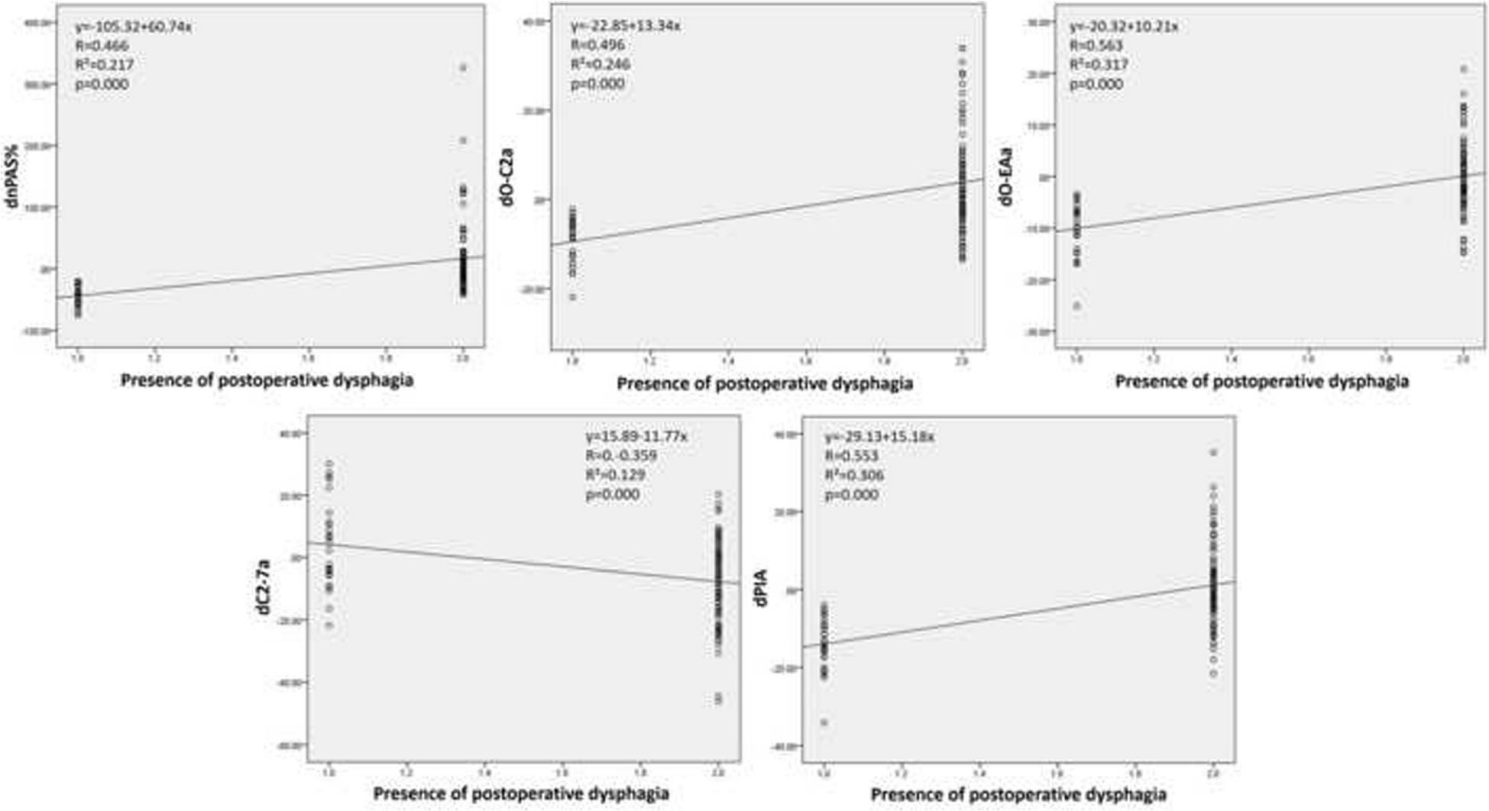
Scatter diagrams between the changes in pre- and postoperative radiographic parameters and the presence of dysphagia. dO-EAa (R2 = 0.317) and dPIA (R2 = 0.306) had a superior correlation with postoperative dysphagia compared with dnPAS%, dO-C2a and dC2-7a

Simple linear regression showed that dnPAS%, dO-C2a, dO-EAa, dC2-7a and dPIA all had significant correlations with the presence of postoperative dysphagia. The scatter diagram and data are shown in Fig. 2.

**Fig. 3:**
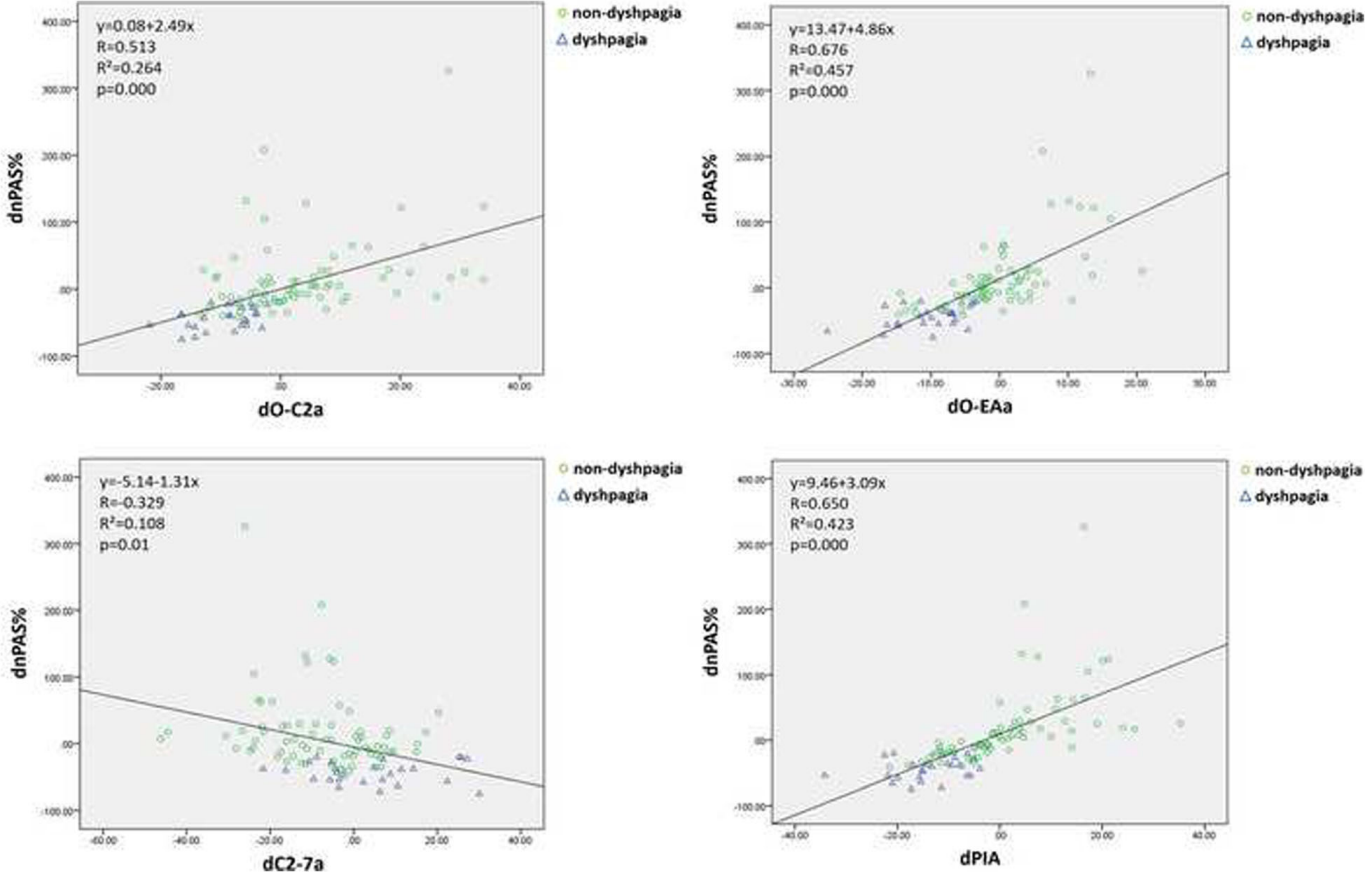
Scatter diagrams between dnPAS% and predictive radiographic parameters. dO-EAa (R2 = 0.457) and dPIA (R2 = 0.423) both have obvious correlations with dnPAS%, superior to those with dO-C2a and dC2-7a

When analyzing the relationship between dnPAS% and other parameters, we found significant correlations in four of them, including dO-C2a, dO-EAa, dC2-7 and dPIA. The R^2^ values of dO-EAa (R^2^=0.457) and dPIA (R^2^=0.423) were similar, and both were above 0.4, which demonstrated a strong correlation with dnPAS%. The scatter diagram and data are shown in Fig. 3. Furthermore, multiple regression analysis showed that among these four parameters, dO-EAa and dPIA were the only significant predictors for dnPAS% (dO-EAa: β=0.435, *p*=0.000; dPIA: β=0.285, *p*=0.029) (Table 3).

**Table 3:** Multivariate linear regression analysis for the influence of predictors on dnPAS%

	Unstandardized coefficient		Standardized coefficient	95% Confidence interval			
Predictors for dnPAS%	B	SE	β	Lower limit	Upper limit	t value	*p*
dO-C2a	0.200	0.531	0.041	-0.854	1.254	0.376	0.708
dO-EAa	3.126	0.729	0.435	1.678	4.574	4.287	0.000*
dC2-7a	-0.256	0.325	-0.064	-0.902	0.390	-0.787	0.433
dPIA	1.358	0.613	0.285	0.141	2.574	2.216	0.029*
Constant	11.528	4.797		2.003	21.054	2.403	0.018*

dnPAS%, [postoperative narrowest oropharyngeal airway space (nPAS) - preoperative nPAS]/preoperative nPAS; dO-C2a, the difference between pre- and postoperative O-C2 angle; dO-EAa, the difference between pre- and postoperative occipital and external acoustic meatus to axis angle; dC2-7a, the difference between pre- and postoperative C2-7 angle; dPIA, the difference between pre- and postoperative pharyngeal inlet angleAdjust R^2^=0.501, *p*<0.001*, Indicates statistically significant differences (*p*<0.05)

Based on the calculated parameters, the required sample size (using 0.8 power and 0.05 significance) for our study was 53 (14 patients in the dysphagia group and 39 in the nondysphagia group). In addition, the statistical power for our sample size was 0.97. This showed that our study was powered to detect the difference in parameters affecting postoperative dysphagia.

**Fig. 4:**
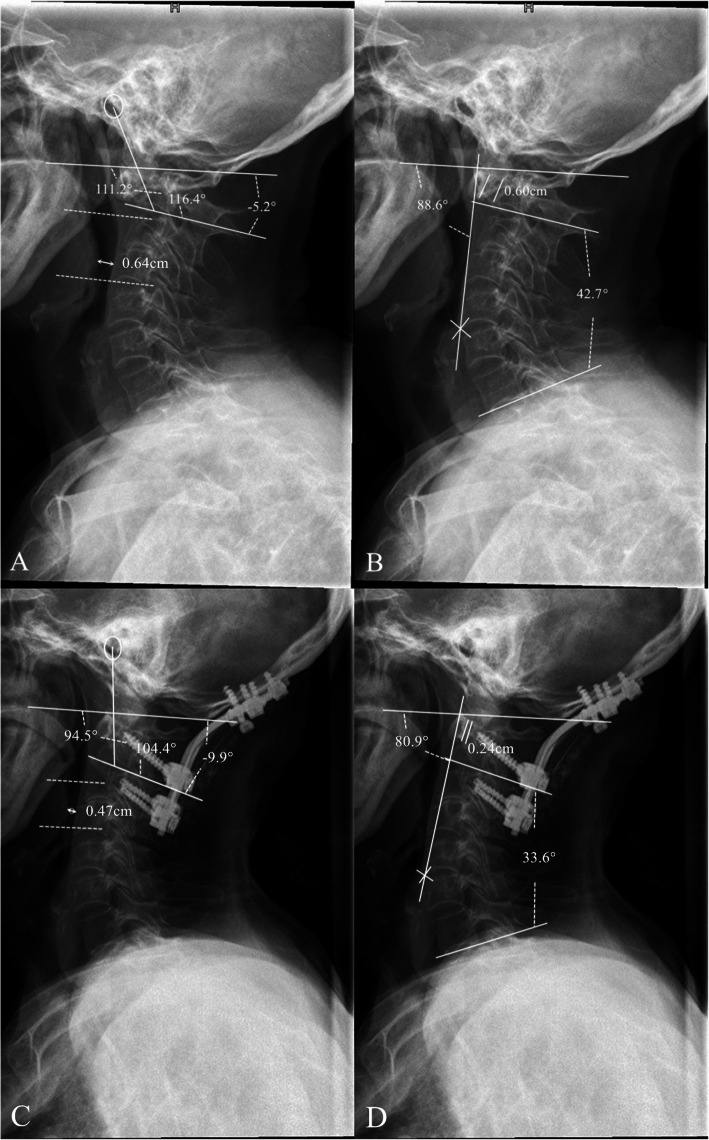
Radiographic presentation of an atlantoaxial dislocation patient suffering postoperative dysphagia. **a** The preoperative values of O-EAa and O-C2a were 111.2° and − 5.2°, respectively, and nPAS was 0.64 cm. **b**The preoperative values of C2-7a and PIA were 88.6° and 42.7°, respectively. ADI was 0.60 cm. **c** and **d** Postoperative measurement of radiographic parameters. On postoperative measurements, O-EAa, O-C2a and C2-7a decreased to 94.5°, -9.9° and 33.6°, respectively, and PIA decreased to 80.9°. Atlantoaxial reduction was achieved. The oropharyngeal space decreased significantly to 0.47 cm and experienced postoperative dysphagia

The radiographic presentation of a patient from the dysphagia group is shown in Fig. 4.

## Discussion

OCF has become a conventional and effective operation method for treating instability of the craniocervical junction caused by trauma, tumors, congenital deformities, rheumatoid arthritis, and infection, et al. [10, 14–16]. It has been reported that 1/6 to 1/4 of patients who underwent OCF experienced dysphagia postoperatively, and only a small proportion of them experienced total symptom relief during follow-up [1–4]. As shown in our study, only two of the 26 dysphagia patients experienced total relief. Thus, risk factors for dysphagia after OCF have been widely discussed [1, 10, 11, 17]. In recent years, how to avoid postoperative dysphagia by adjusting the occipitocervical angle has been widely studied [4, 6–8, 10, 11].

O-C2a has been proven to have predictive ability for post-OCF dysphagia [1, 9, 10, 18]. However, in Izeki et al.’s study, reduction of anterior atlantoaxial subluxation (AAS) caused a decrease in nPAS despite an increase in O-C2a because the atlas shifted posteriorly [11]. Later, Morizane et al. promoted a new parameter, O-EAa, which could reflect not only the change in atlantooccipital angle but also the translational motion of the atlas [7]. They proved that O-EAa was superior to O-C2a in predicting changes in nPAS for AAS patients [19]. In our previous study, we evaluated this angle for all OCF patients and found that the predictive ability of O-EAa surpassed that of O-C2a in postoperative dysphagia [4]. However, during the measurement, we found that the external acoustic meatus was unreadable for some patients. In these cases, the position of the external acoustic meatus was estimated by display of the pinna, which would increase the measurement error. PIA, formed by McGregor’s line and the line linking the center of the C1 anterior arch and the apex of the cervical sagittal curvature, was first promoted by Kaneyama et al. and has been proven to be effective in predicting postoperative dysphagia in patients who underwent occipitospinal fusion. Meanwhile, its anatomic structures can be easily identified on radiographic images [3]. Thus, in our study, we evaluated the predictive ability of PIA in postoperative dysphagia for patients who underwent OCF.

When comparing the radiographic parameters, we found that postoperative O-C2a, O-EAa, PIA and nPAS all had significant decreases in the dysphagia group compared with the nondysphagia group, while C2-7 had a significant increase. When analyzing the linear correlation, the changes in O-C2a, PIA and O-EAa had significant correlations with both dnPAS and the presence of dysphagia. Furthermore, the R^2^ values of O-EAa and PIA were similar and higher than that of O-C2a. The results of multiple regression analysis showed that among all the radiographic parameters, only O-EAa and PIA were significant risk factors for changes in nPAS. Considering all the results, we thought that a decrease in PIA would cause oropharyngeal stenosis and may result in postoperative dysphagia. In addition, PIA has a similar predictive effect as O-EAa in the change in nPAS and the occurrence of postoperative dysphagia.

**Fig. 5:**
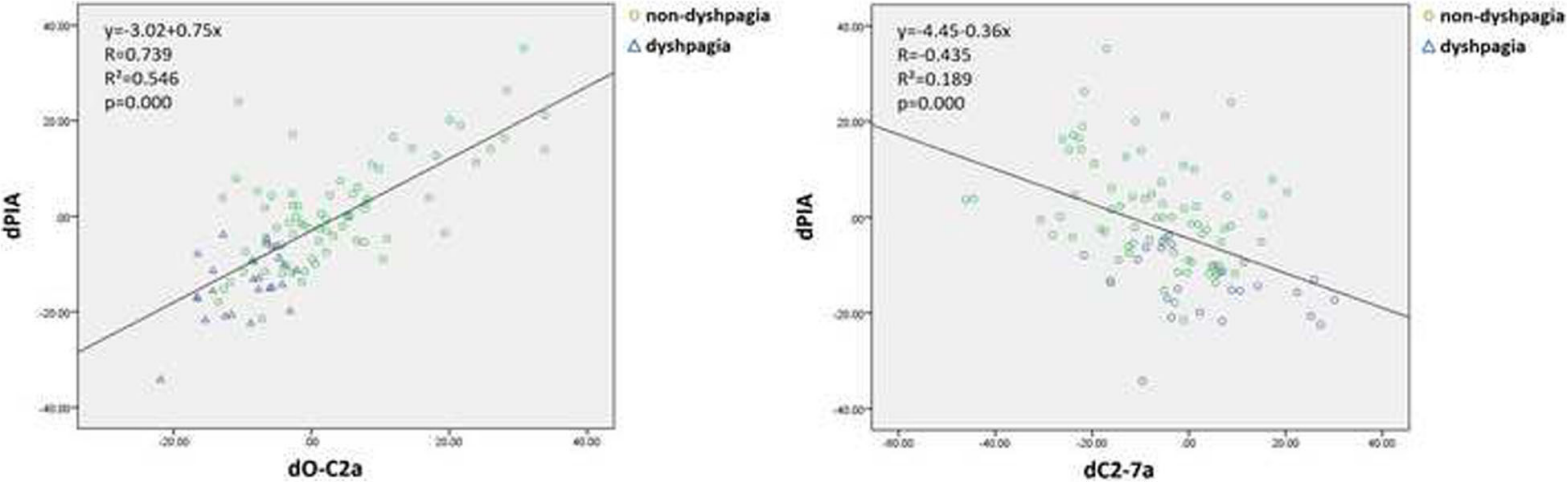
Scatter diagrams between dPIA and predictive radiographic parameters. dO-C2a and dC2-7a could lead to an increase in PIA. In addition, the increase in O-C2a (R2 = 0.546) had a superior correlation with the increase in PIA compared with the decrease in C2-7a (R2 = 0.189)

Previously, Kaneyama et al. considered that PIA was affected by the position of the atlas and cervical sagittal curvature [3]. Thus, in our study, we analyzed the relationships of dADI, dO-C2a, and dC2-7a with dPIA. Similar to their findings, the results of our study showed that dPIA had significant correlations with dO-C2a and dC2-7a, which is either an increase in O-C2a or a decrease in C2-7a could lead to an increase in PIA. Furthermore, the correlation of dO-C2a & dPIA was much higher than that of dC2-7 & dPIA. The scatter diagram and data are shown in Fig. 5. However, the change in ADI had no correlation with the change in PIA (*p*=0.497, R=0.069). We assumed this may be because a reduction in AAS could not only lead to a posterior shift of the atlas but also an increase in O-C2a. The changes had a complicated effect on PIA.

After reviewing the radiographic data, we found that 35 of the 98 patients had a dPIA≥0. All 35 patients had no postoperative dysphagia, and only two of them had a slight decrease in nPAS, which demonstrated that an increase in PIA could help prevent the occurrence of dysphagia. Moreover, we found that the preoperative PIA in the dysphagia group was significantly higher than that in the nondysphagia group. For patients who had a high preoperative PIA, preserving or increasing PIA during surgery sometimes seemed to be difficult due to requirements such as achieving adequate reduction and preserving a horizontal gaze. Thus, for these patients, whether there would be a threshold PIA for preventing postoperative dysphagia was analyzed.

In Kaneyama et al.’s study, no patients experienced dysphagia with a postoperative PIA of no less than 90. They considered PIA≥90 to be a threshold for preventing postoperative dysphagia [3]. In the present study, the sensitivity and specificity of PIA <90° in predicting postoperative dysphagia were 88.5% (23/26) and 58.3% (42/72), respectively. Only three patients with postoperative PIA over 90 experienced dysphagia. The PIAs of these three patients were all close to 90 (90.4, 90.4 and 91.3) and were within the measurement error. Thus, we assume that preserving a PIA over 90 could greatly reduce the occurrence of postoperative dysphagia.

In summary, we believe that for all patients, adjusting a dPIA≥0 during the operation would avert postoperative dysphagia, and for those with a high preoperative PIA in whom a decrease is inevitable, postoperative PIA should be maintained over 90. The surgeon could alter the PIA by adjusting the patient’s head position for an increased O-C2a (through bending the rods or shortening the interval among fixed segments) before the final fixation during surgery.

One major concern about using PIA as the predictor for postoperative dysphagia in OCF patients is that the lower cervical spine was unfixed. The mobile lower cervical spine would change the intraoperative adjustment of PIA. However, theoretically, if we extend the upper cervical spine to increase the O-C2a and PIA before final fixation during surgery, flexion at the lower cervical spine would occur postoperatively to compensate for the horizontal gaze. In this condition, O-C2a maintained while C2-7a decreased, resulting in an increase in the PIA. Therefore, we thought that PIA could be used as a predictor for avoiding postoperative dysphagia in patients who underwent OCF. However, this hypothesis should be proven in future studies.

There are some shortcomings in this study. First, this was a retrospective study; the data collected may be less accurate and complete than prospective studies. Second, the dysphagia of patients was assessed by inquiry, and there was a lack of radiographic evidence, such as the barium swallow test.

## Conclusions

Postoperative dysphagia is a common complication after OCF and affects patients’ daily living. The present study demonstrated that PIA had a similar predictive effect as O-EAa and could be used as a predictor for postoperative dysphagia in patients undergoing OCF. Adjusting intraoperative PIA over preoperative PIA could help avoid post-OCF dysphagia. For those with an inevitable decrease in PIA, preserving the intraoperative PIA more than 90 would to a great extent avert postoperative dysphagia.

## Data Availability

The data that support the findings of this study are available from the corresponding author XY, upon reasonable request. The data are not publicly available due to their containing information that could compromise the privacy of research participants.

## Abbreviations

OCF: Occipitocervical fusion
O-C2a: O-C2 angle
O-EAa: Occipital and external acoustic meatus to axis angle
C2Ta: C2 tilting angle
PIA: Pharyngeal inlet angle
ADI: Atlas-dens interval
C2-7a: C2-7 angle
nPAS: Narrowest oropharyngeal airway space
AAS: Anterior atlantoaxial subluxation
